# Synthesis and Characterization of Alkoxysilane-Bearing Photoreversible Cinnamic Side Groups: A Promising Building-Block for the Design of Multifunctional Silica Nanoparticles

**DOI:** 10.1021/acs.langmuir.2c02472

**Published:** 2022-12-08

**Authors:** Sara Fernanda Orsini, Laura Cipolla, Simona Petroni, Sandra Dirè, Riccardo Ceccato, Emanuela Callone, Roberta Bongiovanni, Sara Dalle Vacche, Barbara Di Credico, Silvia Mostoni, Roberto Nisticò, Luisa Raimondo, Roberto Scotti, Massimiliano D’Arienzo

**Affiliations:** † Department of Materials Science, University of Milano-Bicocca, Via R. Cozzi 55, 20125 Milano, Italy; ‡ Department of Biotechnology and Biosciences, University of Milano-Bicocca, P.za della Scienza 2, 20126 Milano, Italy; § “Klaus Müller” Magnetic Resonance Laboratory, Department of Industrial Engineering, University of Trento, Via Sommarive 9, 38123 Trento, Italy; ∥ Department Industrial Engineering, University of Trento, Via Sommarive 9, 38123 Trento, Italy; ⊥ Department of Applied Science and Technology, DISAT, Politecnico di Torino, Corso Duca degli Abruzzi 24, 10129 Torino, Italy; # Consorzio Interuniversitario Nazionale per la Scienza e Tecnologia dei Materiali, (INSTM), Via G. Giusti, 9, 50121 Firenze, Italy

## Abstract

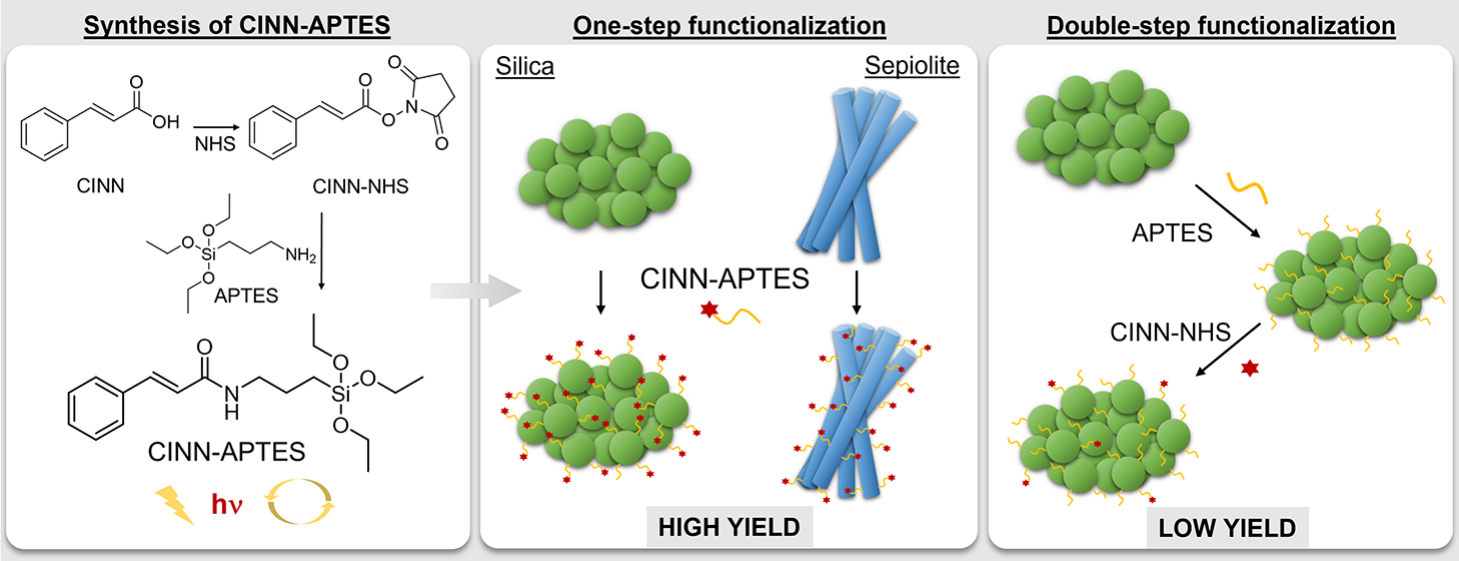

The present study reports on the synthesis of a new alkoxysilane-bearing light-responsive cinnamyl group and its application as a surface functionalization agent for the development of SiO_2_ nanoparticles (NPs) with photoreversible tails. In detail, cinnamic acid (CINN) was activated with *N*-hydroxysuccinimide (NHS) to obtain the corresponding NHS-ester (CINN–NHS). Subsequently, the amine group of 3-aminopropyltriethoxysilane (APTES) was acylated with CINN–NHS leading to the generation of a novel organosilane, CINN-APTES, which was then exploited for decorating SiO_2_ NPs. The covalent bond to the silica surface was confirmed by solid state NMR, whereas thermogravimetric analysis unveiled a functionalization degree much higher compared to that achieved by a conventional double-step post-grafting procedure. In light of these intriguing results, the strategy was successfully extended to naturally occurring sepiolite fibers, widely employed as fillers in technological applications. Finally, a preliminary proof of concept of the photoreversibility of the obtained SiO_2_@CINN-APTES system has been carried out through UV diffuse reflectance. The overall outcomes prove the consistency and the versatility of the methodological protocol adopted, which appears promising for the design of hybrid NPs to be employed as building blocks for photoresponsive materials with the ability to change their molecular structure and subsequent properties when exposed to different light stimuli.

## 1 Introduction

Silica nanoparticles (NPs) represent a powerful platform used in the design of systems employed in a number of applications ranging from catalysis,^1^ separation, and filtration^2^ to optoelectronics,^3^ polymer composites,^4^ sensing devices,^5^ cosmetics,^6^ and biomedical products.^7^ Their high versatility is connected to the possibility of easily modulating the silica bulk properties, including surface morphology, particle size, shape, and porosity, by using a broad variety of synthetic methods.^8^

Furthermore, the surface silanol groups can be easily modified by various organic and inorganic functional groups, altering the surface reactivity and thus leading to specific and unique applications of silica NPs that would otherwise be inaccessible.^9,10^

The recent advent of functional and smart materials showing the ability to change their molecular structure and subsequent properties in response to external stimuli such as light, heat, and pH, has prompted the possibility of modifying the silica surface with photoreversible groups (e.g., coumarin, cinnamyl moieties, stilbene, thymine, and styrylpyrene molecules) endowing these systems with interesting and controllable properties for advanced applications.^11−13^

Different light-induced processes can be exploited, such as isomerization, Diels–Alder cyclization, coordination, disulfide exchange, transesterification, imine formation, and photodimerization reactions. Among them, 2π + 2π cycloaddition reactions mostly involving coumarin derivatives anchored to hybrid NP surfaces have been explored, providing several examples of either potential drug-delivery or light-triggered assembly systems.^11,14,15^

In these approaches, surface functionalization has been achieved by the traditional condensation reaction between silanols and alkoxy- or chloro-silanes bearing coumarin functionalities. For instance, Mal et al.^16^ functionalized the cavity surfaces of MCM-41 mesoporous silica with 7-[(3-triethoxysilyl)propoxy]coumarin, showing that it is possible to control the uptake, storage, and release of organic molecules by photocontrolled dimerization and cleavage of coumarin groups. More recently, Kehrloesser et al.^17^ extended the same strategy toward Stöber silica NPs, studying in detail the photochemistry of coumarin groups at the surface.

Silica functionalization with coumarin units has been also exploited in several studies for activating photocontrolled self-interaction processes. Owing to the reversible photodimerization and photocleavage of coumarin moieties, the generation of light-controlled NP assembly with reversible morphological structures was attained by alternating irradiation cycles with 365 nm and 254 nm light.

In the aforementioned studies, the coumarin-modified organosilanes are generally obtained by introducing into fluorescent dye molecules of allyl terminal groups, which are then converted into trialkoxysilyl groups by hydrosilylation reaction.^18^ Moreover, several approaches also involve the primary modification of silica with amino silanes, such as 3-aminopropyltriethoxysilane (APTES), and the subsequent condensation reaction with coumarin carboxylic derivatives through amide chemistry.^19,20^

Although this double-step procedure with amide bond formation represents a relatively simple and well-established method to anchor biologically relevant molecules, it entails several drawbacks, especially if applied for preparing silica NPs decorated with photoreversible units. In fact, APTES can interact with the silanol/silanolate groups in many possible ways, that is, via hydrogen bonds, electrostatic attractions, and siloxane bonds, possibly resulting in low silane grafting density or weakly attached silane molecules, with a consequent loss of anchoring sites for the functional molecule.^21−23^

Moreover, an excess amount of APTES is usually required for initial surface modification, with consequent generation of oligomeric layers and of unreacted amine groups in the final materials, since the secondary grafting reactions are often incomplete. The remaining free amine groups can further impart instability and a gradual desorption of aminopropyl-silane moieties from silica, as a consequence of self-catalyzed amine-mediated hydrolysis.^24,25^

In this scenario, it would be worthwhile to develop a new synthetic strategy to prepare silica NPs, by a one-step reaction between the NPs and the alkoxysilane possessing already in place the desired photoreversible unit; the reaction should occur in a controlled manner under mild conditions and to enable a remarkable surface functionalization yield.

Taking up this challenge, the present study proposes an original synthetic protocol to obtain a new alkoxysilane-bearing covalently bound photoreversible cinnamyl functionalities, which has been then anchored, by a simple one-step procedure, to silica and silicate particles, leading to the design of photo-responsive nanomaterials.

To assemble the novel silane, *trans*-cinnamic acid (CINN) was selected as an aromatic carboxylic acid naturally appearing in the plant kingdom. This secondary metabolite plays key physiological roles in plant growth, development, reproduction, and disease resistance, and it has also been used by medicinal chemists to alter the potency, permeability, solubility, or other parameters of a selected drug or pharmacophore.^26^ In addition, the cinnamyl group is one of the moieties reported to undergo photodimerization that can be reversed upon application of an appropriate light wavelength.^27^

As widely reported, CINN possesses −COOH carboxyl head groups with the ability to bind NH_2_ groups present in APTES molecules. This reaction usually requires acid or basic conditions, which often results in the polycondensation of organosilane, hampering the obtaining of the product.^28^ Thus, to prevent these downside effects and to ensure the generation of covalent bonding, the COOH groups of CINN were activated by *N*-hydroxysuccinimide (NHS) in aprotic solvents by a reaction between the acid and a carbodiimide (*N*,*N*′-dicyclohexylcarbodiimide, DCC) to form a NHS-ester suitable for the amide condensation reaction.

Upon confirmation of the product identity by NMR spectroscopy, the novel organosilane (CINN-APTES) has been used for functionalizing silica (SiO_2_@CINN-APTES) NPs.

The structural, surface, and morphological features of functionalized SiO_2_ NPs were extensively investigated by solid-state NMR, attenuated total reflection Fourier transform infrared (ATR-FTIR) spectroscopy, and scanning and transmission electron microscopies (SEM and TEM). The functionalization degree was carefully estimated by thermogravimetric (TGA) and CHNS elemental analysis, and the results were compared with those obtained by the double-step conventional post-grafting procedure.

To prove the consistency and the versatility of the proposed synthetic strategy, the functionalization of naturally occurring sepiolite (Sep) fibers, widely employed as filler in many technological applications, was also accomplished.

Finally, the photochemical properties of the obtained SiO_2_@CINN-APTES system have been carefully checked by UV diffuse reflectance (UV-DRS).

The overall experimental data indicate the efficacy of the adopted synthetic strategy in providing tailored silica and silicate NPs with photoreversible tails, which can be potentially used in drug release applications or as building blocks for innovative organic–inorganic hybrid materials with peculiar self-healing or recyclability properties.

## 2 Experimental Section

### 2.1 Materials

*N*-Hydroxysuccinimide 98% (NHS) and *N*,*N*′-dicyclohexylcarbodiimide 99% (DCC) were purchased from Sigma-Aldrich and used as received. APTES 98% was purchased from abcr. Ammonia solution 25% for analysis was purchased from Merck-Millipore.

Tetrahydrofuran (THF) 99% and anhydrous THF 99.8+% were purchased from Alfa Aesar. Ethanol (absolute) and acetonitrile (MeCN, HPLC grade) were purchased from VWR; methanol (HPLC grade), and sodium hydroxide 98% (NaOH) were purchased from Thermo Fisher Scientific. SiO_2_ NPs were synthesized by a modified Stöber process in previous work, and sepiolite nanofibers were purchased from the Pangel S9—Tolsa group. *trans*-Cinnamic acid (CINN) was purchased from Merck-Millipore and recrystallized in acetone according to a previously reported procedure.

### 2.2 Synthesis of CINN–NHS Ester

CINN (1.00 g, 6.75 mmol) was dissolved in 14 mL of a solution of MeCN/anhydrous THF (1:1) under magnetic stirring. DCC (1.809 g, 8.77 mmol) and NHS (0.854 g, 7.42 mmol) were added to the solution, and the reaction was kept under magnetic stirring at room temperature for 24 h. The resulting suspension was filtered, and the organic phase was concentrated. The white residue was resuspended in 10 mL of MeOH, and the suspension left under stirring for 30 min at RT. The precipitate was collected by filtration, washed with MeOH (4 × 10 mL), and dried under vacuum overnight. The yield of the crude material was about 55%. ^1^H NMR (400.13 MHz, CDCl_3_): δ 7.98 (d, *J* = 16.05 Hz, 1H), 7.63 (dd, *J* = 7.66, 1.62 Hz, 2H), 7.50–7.37 (m, 3H), 6.65 (d, *J* = 16.05 Hz, 1H), 2.88 (s, 4H). NMR data are consistent with the literature data.^29^

### 2.3 Synthesis of CINN-APTES

In a one-neck round-bottom flask, CINN–NHS (100 mg, 0.41 mmol) was dissolved in anhydrous THF (4.1 mL). APTES (96 μL, 0.41 mmol) was added slowly dropwise, and the reaction was kept at RT for 1 h. The solution was evaporated yielding a colorless oil constituting the CINN-APTES product. The peculiar reactivity of the new organosilane does not allow purification, and the product was used as is for the functionalization of silica NPs. The yield of organosilane, calculated from ^1^H NMR, was about 90%. ^1^H NMR (400.13 MHz, CDCl_3_): δ 7.65 (d, *J* = 15.65 Hz, 1H), 7.52 (dd, *J* = 7.60, 1.84 Hz, 2H), 7.41–7.33 (m, 3H), 6.41 (d, *J* = 15.65 Hz, 1H), 5.99 (s, 1H), 3.87 (q, *J* = 6.99 Hz, 6H), 3.44 (q, 6.54 Hz, 2H), 2.64 (s, 4H), 1.76–1.66 (m, 2H), 1.27 (t, *J* = 6.98 Hz, 9H), 0.72 (t, *J* = 8.00 Hz, 2H). ^13^C NMR (400.13 MHz, CDCl_3_): δ 165.89, 140.74, 134.94, 129.55, 128.78, 121.71, 120.86, 58.51, 42.00, 25.59, 22.86, 18.29, 7.82.

### 2.4 One-step Preparation of CINN-APTES-Functionalized Silica NPs

In a two-neck round-bottom flask, SiO_2_ NPs (100 mg) were dispersed in 3.0 mL of EtOH by ultrasonic bath (2 min) and the mixture was brought under reflux conditions. A solution of NH_3_ 25 wt % (10 μL) and the proper amount of CINN-APTES dissolved in 2.0 mL of EtOH were added dropwise to the dispersion, and the reaction mixture was kept under reflux condition and stirred vigorously for 12 h. The silane (CINN-APTES) concentration has been established as a function of the number of surface Si–OH groups of SiO_2_ NPs (i.e. 13.67 Si–OH/nm^2^ from TGA analysis), considering that usually only 2/3 of the ethoxy groups of alkoxysilanes undergo the condensation reaction with surface silanol terminations. In the present conditions, this means Si–OH/ethoxy groups of CINN-APTES ratio of 1.5:3 (i.e. 1:2).

Finally, the mixture was centrifuged at 9000 revolutions per minute (rpm) for 15 min and washed several times with EtOH to remove excesses of CINN-APTES and NHS (by-product of CINN-APTES synthesis). Functionalized SiO_2_ NPs (SiO_2_@CINN-APTES) were finally dried under vacuum for 3 h.

### 2.5 Double-Step Preparation of CINN-APTES-Functionalized Silica NPs

#### 2.5.1 Step1: Functionalization of Silica NPs with APTES

In a two-neck round-bottom flask, SiO_2_ NPs (200 mg) were dispersed in 10.0 mL of EtOH by ultrasonication (2 min), and the mixture was brought under reflux conditions. A solution of NH_3_ 25 wt.% (20 μL) and APTES (218 μL) were added dropwise to the dispersion, and the reaction mixture was kept under reflux conditions and stirred vigorously for 12 h. Finally, the mixture was centrifuged at 9000 rpm for 15 min and washed several times with EtOH. Finally, SiO_2_@APTES NPs were dried under vacuum for 3 h.

#### 2.5.2 Step 2: Derivatization of the Amino Group of SiO_2_@APTES

SiO_2_@APTES NPs (100 mg, 0.053 mmol of NH_2_, 1 equiv) were dispersed in 5.0 mL of THF in a two-neck round bottom flask by ultrasonication (2 min). Then, CINN–NHS was added to the dispersion, and the reaction was kept under magnetic stirring at room temperature for 1 h. In the present case, since the silica surface is already modified with APTES, the amount of CINN–NHS added (20 mg, 0.082 mmol, 1.5 equiv) has been calculated as a function of the number of −NH_2_ groups (see Table 1), in order to have a −NH_2_/CINN–NHS group ratio of 1:1.5. Thus, the ratio between the number of anchoring groups and the cinnamic units is almost the same utilized in the one step strategy.

**Table 1:** Functionalization Degree with APTES and CINN-APTES Units over SiO_2_ NPs by One-Step and Two-Steps Procedures, Calculated from TGA (According to Eqs S1 and S2) and CHNS Analysis

	σ (no. molecules/nm^2^)	
	TGA	CHNS
SiO_2_@APTES NPs	1.77	1.44
SiO_2_@APTES@CINN NPs (double-step)	0.24	0.32
SiO_2_@CINN-APTES NPs (one-step)	1.82	1.86

Finally, the suspension was centrifuged at 9000 rpm for 15 min and washed several times with THF. Derivatized SiO_2_ NPs (SiO_2_@CINN@APTES) were dried overnight in open air.

### 2.6 Pre-treatment of Sepiolite Nanofibers (Sep)

In a one-neck round-bottom flask, Sep (10.0 g) was dispersed in 500 mL of 0.01 M NaOH, and the suspension was vigorously stirred at RT for 24 h. Then, the mixture was centrifuged at 9000 rpm for 15 min and the product (Sep-OH) was washed several times with deionized H_2_O up to pH 7 of supernatant and dried in open air for 3 days. The nanofibers were grinded and dispersed in 100 mL of deionized H_2_O and then freeze-dried.

### 2.7 Synthesis of CINN-APTES-Functionalized Sep-OH Nanofibers

In a one-neck round-bottom flask, CINN–NHS (100 mg, 0.41 mmol) was dissolved in anhydrous THF (4.1 mL). APTES (96 μL, 0.41 mmol) was added dropwise, and the reaction was kept at RT for 1 h, in order to obtain the CINN-APTES silane. Then, pre-treated sepiolite (Sep-OH, 24.7 Si–OH groups/nm^2^ from TGA analysis) nanofibers (55 mg) were dispersed in the reaction environment, and the mixture was brought and kept under reflux condition and stirred vigorously for 24 h.

Finally, the product (Sep-OH@CINN-APTES) was recovered by centrifugation at 9000 rpm for 15 min, washed several times with THF to remove excesses of CINN-APTES and NHS (sub-product of CINN-APTES synthesis), and dried under vacuum for 3 h.

### 2.8 Materials Characterization

Liquid-state NMR analyses were performed on samples dissolved in CDCl_3_ using a Bruker Avance 400 WB spectrometer operating at a proton frequency of 400.13 MHz.

Solid-state nuclear magnetic resonance (ss-NMR) analyses were carried out with a Bruker 400WB spectrometer operating at a proton frequency of 400.13 MHz with cross-polarization pulse sequence under the following conditions: ^29^Si frequency: 79.48 MHz, contact time 5 ms, decoupling length 5.9 μs, recycle delay 10 s, 1k scans. ^13^C frequency: 100.48 MHz, contact time 2 ms, decoupling length 5.9 μs, recycle delay: 3 s, 5k scans. Samples were packed in 4 mm zirconia rotors, which were spun at 8 kHz, under airflow. Adamantane and Q_8_M_8_ were used as external secondary references. The Si structural units are labeled according to the usual NMR notation: T^*n*^ and Q^*n*^ indicate R-SiO_3_ and SiO_4_ silicon units, respectively, and *n* is the number of oxo-bridges.

Bruker TopSpin software was used for the lineshape analysis. The results were considered acceptable with a confidence level >95%.

Fourier transform infrared (FTIR) spectra were measured in attenuated total reflectance (ATR) mode at room temperature in the range of 4000–550 cm^–1^ with a ThermoFisher Nicolet iS20 with a spectral resolution of 4 cm^–1^ and 64 scans. The ATR-FTIR spectra were analyzed by OMNIC software and reported after background subtraction and baseline correction.

TGA thermograms were collected by using a Mettler Toledo TGA/DSC1 STARe system at a constant air flow (50 cm^3^ min^–1^). The materials were heated from 30 to 150 °C at the rate of 10 °C min^–1^, with an isothermal step at 150 °C for 10 min, followed by heating from 150 to 1000 °C at the rate of 10 °C min^–1^, keeping the samples at 1000 °C for 5 min.

SEM images on materials were collected by a Vega TS5136 XM Tescan microscope in a high-vacuum configuration. The electron beam excitation was 30 kV at a beam current of 25 pA, and the working distance was 12 mm. In this configuration, the beam spot was 38 nm. The samples were dispersed in EtOH, deposited onto an aluminum substrate by drop-casting and covered with gold coating.

TEM images on materials were collected by using a JEOL JEM-2100Plus TEM (JEOL, Akishima, Tokio, Japan) operating at an acceleration voltage of 200 kV, equipped with an 8-megapixel Gatan (Gatan, Pleasanton, CA, USA) Rio complementary metal-oxide-semiconductor camera. The samples were deposited onto carbon-coated Cu TEM mesh grids by drop-casting dilute NPs dispersions in ethanol.

Diffuse reflectance spectroscopy was performed using a PerkinElmer, precisely, Lambda 1050+ UV/vis/NIR spectrophotometer. The powders were dispersed in EtOH and drop-casted on a quartz slide.

UV–vis spectra of samples dissolved in CHCl_3_ were acquired using a UV–vis Cary 60 spectrophotometer, using a 5 mm cuvette.

The elemental composition was analyzed by using CHNS Analyzer Elementar VarioMICRO.

The photochemical reactivity of CINN was assessed by using a UVLS-24 Fisher UV-lamp equipped with 6 W lamp emitting at 365 and 254 nm and monitored by ATR-FTIR and UV–vis spectroscopies. In detail, photodimerization was performed by irradiating the sample powders at 365 nm for 24 h, while the photocleavage was performed under UV irradiation at 254 nm for 20 days. In both cases, the sample powders were at 3–4 cm from the UV lamp.

## 3 Results and Discussion

### 3.1 Photoreversible Properties of Re-crystallized CINN

Although widely reported in the literature,^27,30^ the photochemical cross-linking behavior of the re-crystallized *trans*-cinnamic acid was preliminary verified by ATR-FTIR and UV–vis spectroscopy. The results are summarized in Figure S1 (see Supporting Information).

Several striking differences can be observed between the FTIR spectrum of CINN and that recorded after UV irradiation at 365 nm (Figure S1a). In detail, the generation of two intense bands at 1750 and 1700 cm^–1^, probably associated to carbonyl groups occurring in different dimeric forms of cinnamic acid units (e.g., truxillic acid or truxinic acid units generated from head-to-tail and head-to-head monomer interactions, inset in Figure S1), is clearly visible in the spectrum of CINN after UV irradiation at 365 nm for 24 h. Moreover, a significant depletion of the C=C vibration at 1630 cm^–1^ is detectable. These results suggest that an almost complete conversion to the dimeric forms has occurred.^30^ Subsequent UV irradiation at 254 nm leads to a very slow but progressive depletion of the bands related to the dimeric species, envisaging a gradual recovery of the initial structure.

The photodimerization of CINN units was also monitored by UV–vis spectroscopy. In detail, spectra were recorded on a solution (5 × 10^–5^ M) of CINN in chloroform as a function of time upon irradiation at λ ≥ 365 nm (Figure S1b). A sharp decrease of the absorption band at ∼275 nm indicated the successful cycloconversion of the cinnamic units, as widely reported in the literature.^31^

### 3.2 Synthesis and Characterization of CINN-APTES

**Scheme 1:**
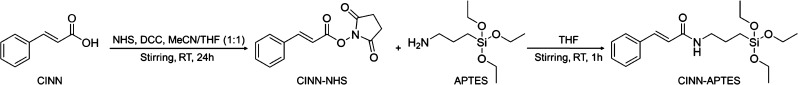
Preparation of CINN-APTES

The preparation procedure of the new CINN-APTES organosilane is described in Scheme 1.

In the first step, NHS ester of CINN was synthesized following a revised procedure proposed by Kozlov et al.^29^ To compare the chemical structure of the obtained CINN–NHS to that reported in the literature, liquid state ^1^H NMR, ^13^C NMR, and ^1^H–^13^C HSQC NMR analyses were performed (Figures S2–S4). As expected, the molecule shows the same structural characteristics of the system reported in the literature.^29^

**Figure 1:**
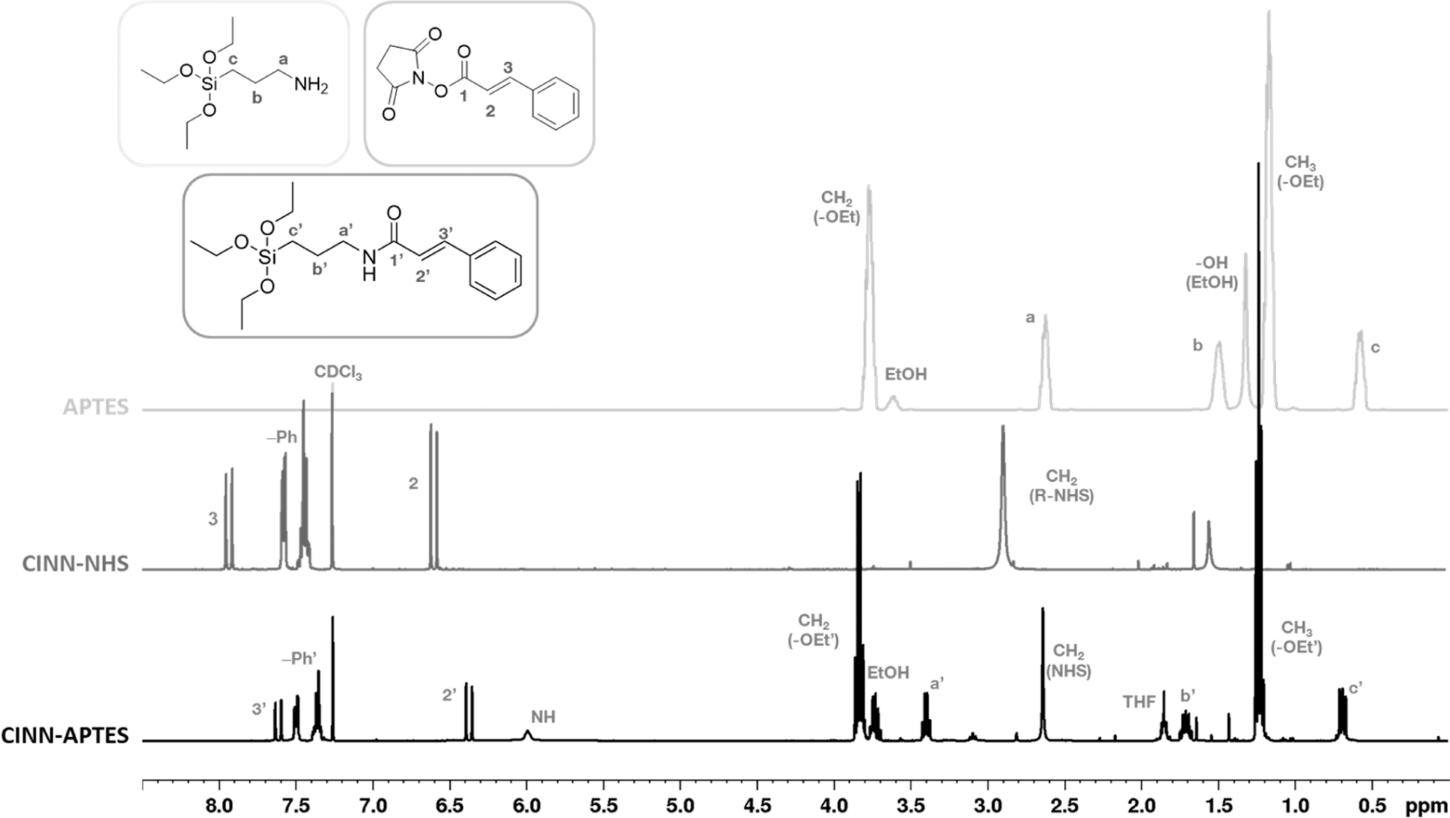
^1^H NMR spectra of APTES, CINN–NHS ester, and the novel CINN-APTES organosilane.

CINN–NHS was then reacted with APTES to obtain CINN-APTES. The structure of the new organosilane was assessed by ^1^H NMR characterization (Figure 1). Spectra of CINN–NHS and APTES were also collected and reported for comparison.

An upfield shift of the peaks in the region between 8.00 and 6.00 ppm with respect to CINN–NHS can be observed for CINN-APTES, indicating a modification of the chemical environment of the vinyl aryl protons. The disappearance of the peak at 2.62 ppm (a in APTES) and the appearance of the peak at 3.4 ppm (a′ in CINN-APTES) instead witness the loss of the amine group and the formation of an amide group. In addition, the ratio of the signal area between a′ and 3′ is equal to two, in line with the ratio of protons of the two relative groups in the molecule. Finally, the upfield to 2.64 ppm of the CH_2_(NHS) resonance indicates the presence of free NHS, as a side-reaction product. These results confirm the synthesis of a new trialkoxysilane bearing light-responsive cinnamyl group. Detailed ^1^H NMR, ^13^C NMR, and ^1^H−^13^C HSQC NMR spectra are reported in Figures S5–S7 in Supporting Information.

In order to verify the absorption features of novel silane, the UV–vis spectrum was acquired and compared to that of the parental CINN–NHS molecule (Figure S8). The wavelength of the maximum absorption of CINN-APTES and CINN–NHS corresponds to 277 and 282 nm, respectively. The small hypsochromic shift in the absorption band for CINN-APTES may be reasonably related to the modification of the cinnamic group structure.

### 3.3 CINN-APTES-Functionalized Silica NPs and Sep Nanofibers

**Figure 2:**
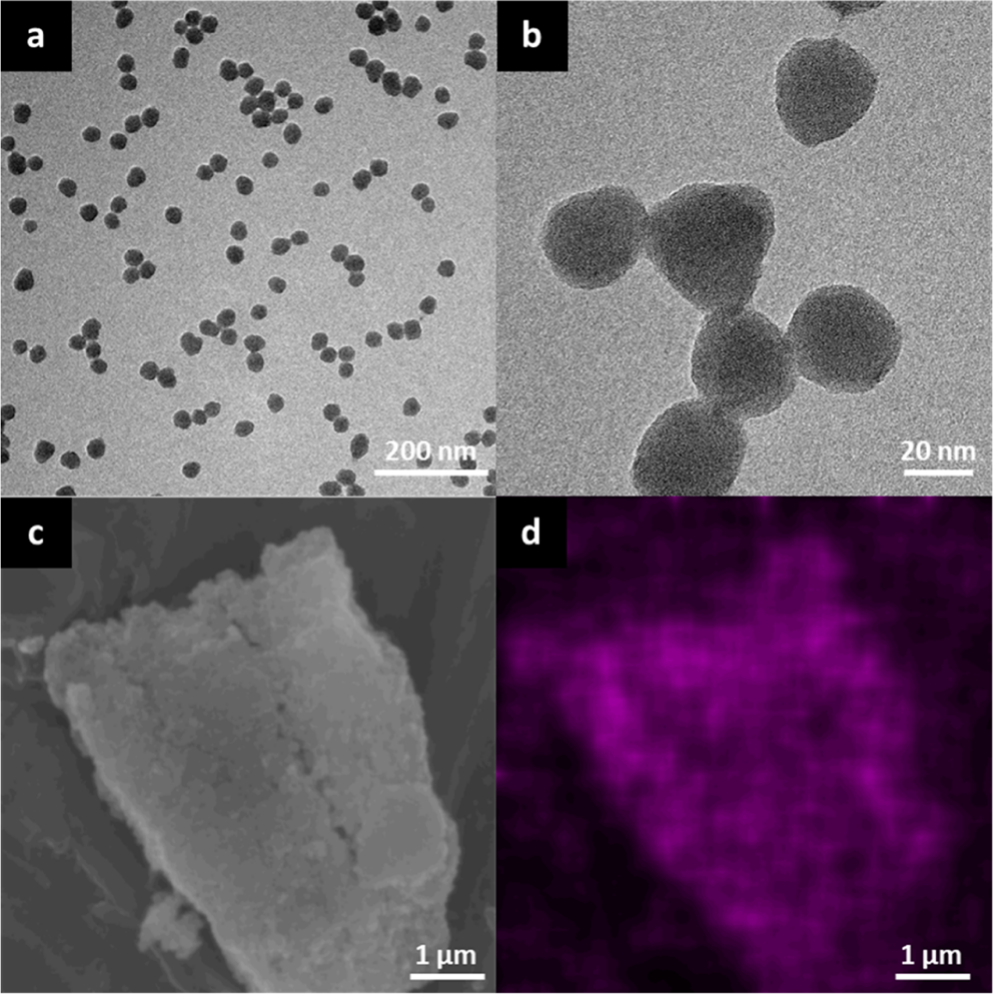
(a,b) TEM images of SiO_2_ NPs; (c) SEM micrograph of SiO_2_@CINN-APTES agglomerate; and (d) corresponding EDX C elemental map. Figure 3 displays the comparison between infrared spectra of CINN-APTES, bare SiO_2_, and SiO_2_@CINN-APTES NPs.

At first, the morphological features of bare silica NPs were investigated by TEM microscopy (Figure 2a,b). The presence of nanospheres with homogeneous dimensions (average diameter of 31 ± 3 nm) can be observed. The BET specific surface area, estimated in a previous work,^32^ is 272.7 ± 0.6 m^2^ g^–1^.

The grafting of CINN-APTES at the silica surface was preliminarily inspected by SEM–EDX analysis.

In detail, Figure 2c,d shows SEM and SEM–EDX micrographs of the same SiO_2_@CINN-APTES NPs agglomerate, respectively. The distribution of carbon atoms (pink dots in Figure 2d) appears homogeneous and located at the surface, probing the anchoring of the CINN-APTES units on the silica substrate.

**Figure 3:**
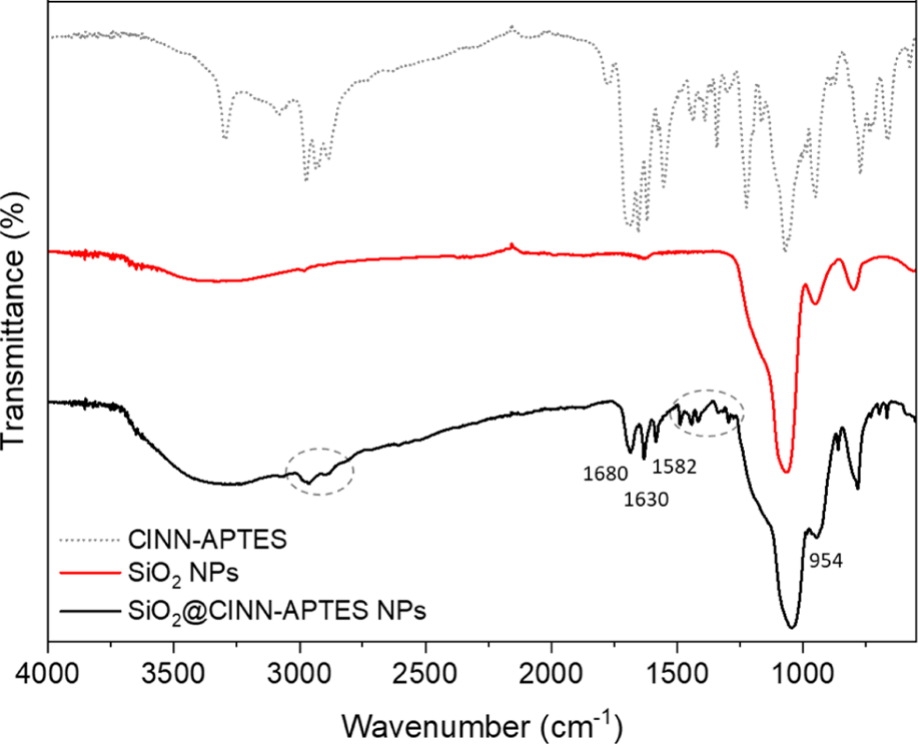
ATR-FTIR-normalized spectra of pristine SiO_2_ NPs (red solid line), CINN-APTES novel organosilane (dashed grey line) and SiO_2_@CINN-APTES NPs (black solid line).

**Figure 4:**
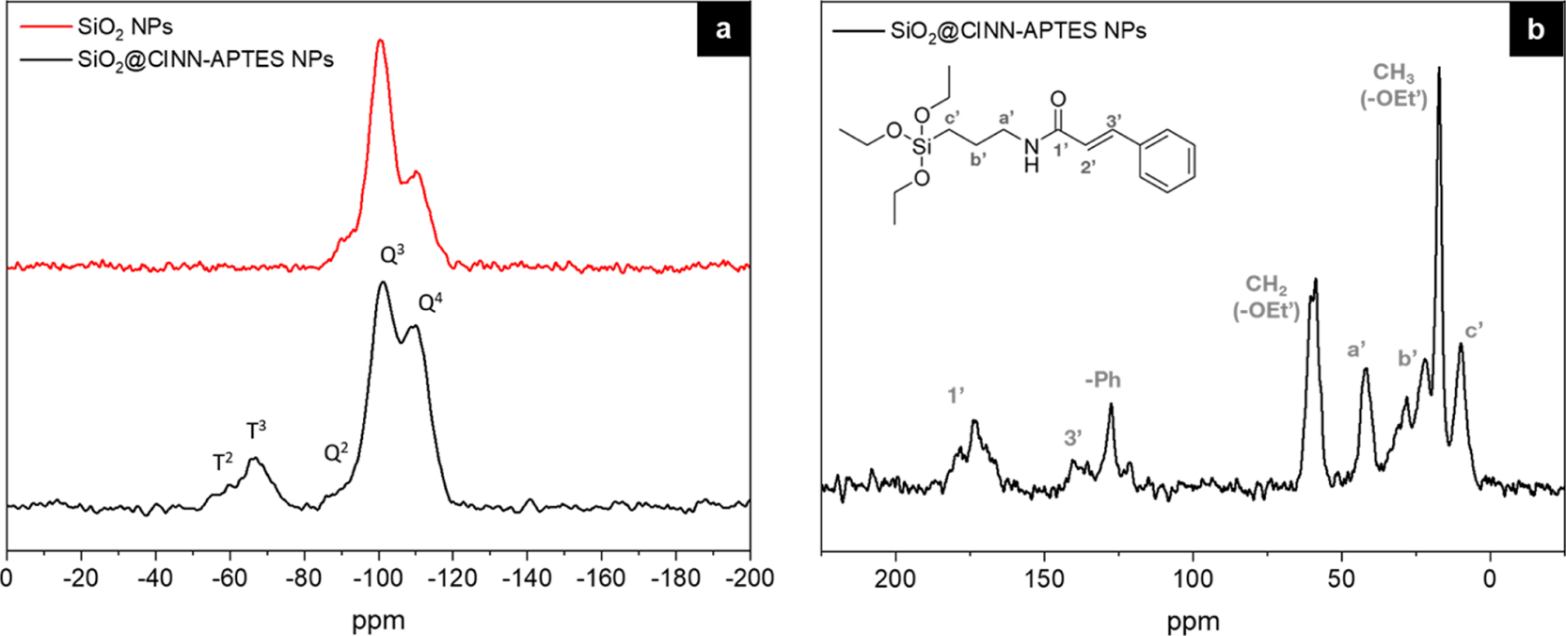
(a) ^29^Si CPMAS NMR spectra of SiO_2_ NPs (red line) and SiO_2_@CINN-APTES NPs (black line) and (b) ^13^C CPMAS spectrum of SiO_2_@CINN-APTES NPs.

The surface modification of SiO_2_ with CINN-APTES NPs was further assessed by ATR-FTIR and solid-state NMR (Figures 3 and 4).

The spectrum of SiO_2_ NPs shows the following main bands: the stretching and the bending vibrational modes of OH groups from physically absorbed water, respectively, in the region between 3700 and 3200 cm^–1^ and at 1633 cm^–1^; the vibrations of Si–O–Si stretching at 1060 cm^–1^; finally, the Si–OH stretching at 954 cm^–1^. In the case of SiO_2_@CINN-APTES, the peaks in the region of 3000 cm^–1^ are due to the C–H asymmetric stretching vibrations of the aliphatic chain and aromatic ring of CINN-APTES. The characteristic bands of vibrational stretching C=O (1680 cm^–1^) of the amide group and of C=C conjugated to an aromatic ring (1630 cm^–1^) indicates the occurrence of CINN-APTES at the silica surface. In addition, the peak at 1582 cm^–1^ is related to the N–H bending of the amide group and to the C–C stretching of the aromatic ring, while the bands between 1550 and 1350 cm^–1^ belong to the C–C stretching vibrations of the aromatic ring of CINN-APTES. Finally, the depletion of the Si–OH stretching at 954 cm^–1^ indicates a decrease of Si–OH groups, which is an index of their involvement in the grafting process. These results qualitatively assess the presence of CINN-APTES at the silica surface.

^29^Si and ^13^C solid-state NMR investigations allowed us to validate the generation of covalent bonds upon functionalization of SiO_2_ NPs with the new organosilane (Figure 4). The ^29^Si CPMAS spectrum of pristine SiO_2_ NPs (red line in Figure 4a) displays the typical signals of Q^4^, Q^3^, and Q^2^ units, respectively, at −110, −100, and −92 ppm.^29^ SiO_2_@CINN-APTES shows the same Q resonances, together with the T^3^ and T^2^ signals at −66 and −57 ppm related to the grafted APTES molecules (black line in Figure 4a). From a qualitative point of view, it can be noticed that the functionalization with the organosilane increases the condensation degree of the silica network, according to the increase of Q^4^ resonance at the expense of Q^2^ and Q^3^ units.

The results of the quantitative analysis (Table S1) performed on ^29^Si MAS spectra (not shown) reveal that for every 100 Q units, there are almost 6 T units. The silica NPs show a good condensation degree (DOC) that increases with the subsequent functionalization, as observed in the CPMAS spectrum.

Figure 4b shows the ^13^C CPMAS spectrum of the SiO_2_@CINN-APTES sample, which clearly displays the fingerprint of the organic groups of CINN-APTES. Besides a not negligible presence of unreacted −OEt units, the features of the carbon resonance in the propyl chain give information on the effective organosilane grafting onto silica NPs. In detail, the downfield position of the c′ peak (Si–CH_2_– methylene carbon) indicates condensation of the inorganic head, and b′ carbon resonance is split into two components at 28.1 and 22.1 ppm, in consequence of the sensitivity of b′ carbon to the electronic environment of terminal nitrogen, which is affected by of the structural rearrangements and propyl chain orientation (γ-gauche effect) due to interaction with particles’ surface.^33,34^ In addition, the line shape of the amide bond 1′ is asymmetric, with at least two components, probably reflecting the different conformations assumed, as already suggested by the split of resonance b′.

The overall results are complementary to the ATR-FTIR ones and definitively assess the modification of silica surfaces with CINN-APTES molecules through the generation of covalent bonds.

**Figure 5:**
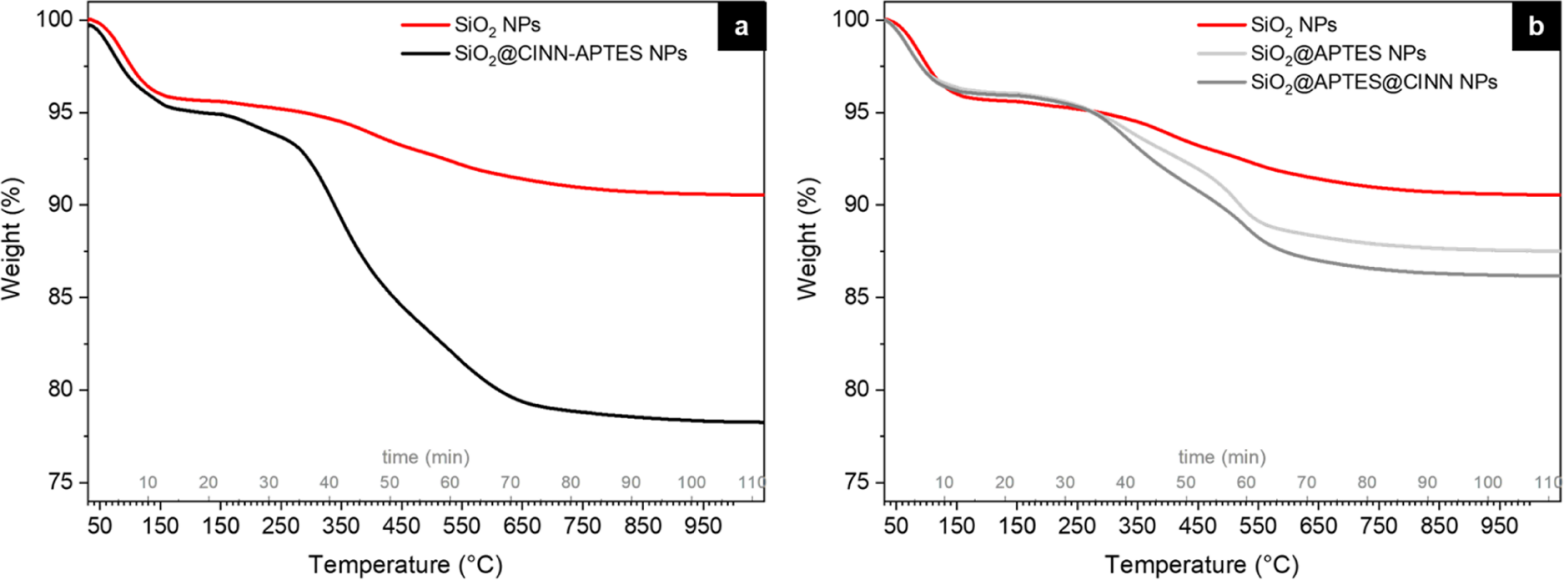
TGA profiles of pristine SiO_2_ NPs and (a) SiO_2_@CINN-APTES NPs, (b) SiO_2_@APTES, and SiO_2_@APTES@CINN NPs.

To quantitatively determine CINN-APTES functionalization degree, TGA and CHNS analyses were performed on SiO_2_@CINN-APTES and on pristine silica NPs. From the thermal profiles (Figure 5a), the reaction yield of SiO_2_ functionalization with CINN-APTES and the number of molecules over the SiO_2_ surface (σ, molecules/nm^2^) were estimated (according to eqs S1 and S2 in the Supporting Information) and are reported in Table 1.

Moreover, to evaluate and compare the efficacy of the one-step modification approach, the functionalization degree was also assessed for SiO_2_@APTES@CINN NPs prepared by the double-step conventional post-grafting procedure reported in Scheme S1 (see Supporting Information).

By this route, silica NPs were initially functionalized with APTES (SiO_2_@APTES NPs) using the same reaction conditions of the synthesis of SiO_2_@CINN-APTES. Then, the amino group of APTES grafted on the silica surface was modified with CINN–NHS to give SiO_2_@APTES@CINN NPs.

From the TGA profiles (Figure 5b) and using the same equations reported in Supporting Information, the σ for each sample was calculated and reported in Table 1.

The functionalization degree was also estimated by CHNS analysis, from the weight percentages of carbon atoms in bare SiO_2_, SiO_2_@APTES, SiO_2_@CINN-APTES, and SiO_2_@APTES@CINN NPs (see details in Supporting Information). The obtained values well match with those retrieved from TGA (Table 1).

These results unveil a much better functionalization efficacy by the one-step route compared to the double-step one. In fact, as shown in Table 1, the one-step procedure allows to bind onto silica surface, a number of molecules per nm^2^ six time higher that that obtained by the double-step one, suggesting that the direct use of the novel CINN-APTES organosilane prevents the usual drawbacks associated with the post-grafting of silica surfaces already pre-functionalized with APTES, that is possible hydrolysis and decomposition before further modification. Furthermore, in the double-step procedure, the amino group of APTES could also be involved in an electrostatic interaction with superficial silanols of NPs, with a consequent loss of anchoring sites for the functional molecules.

**Figure 6:**
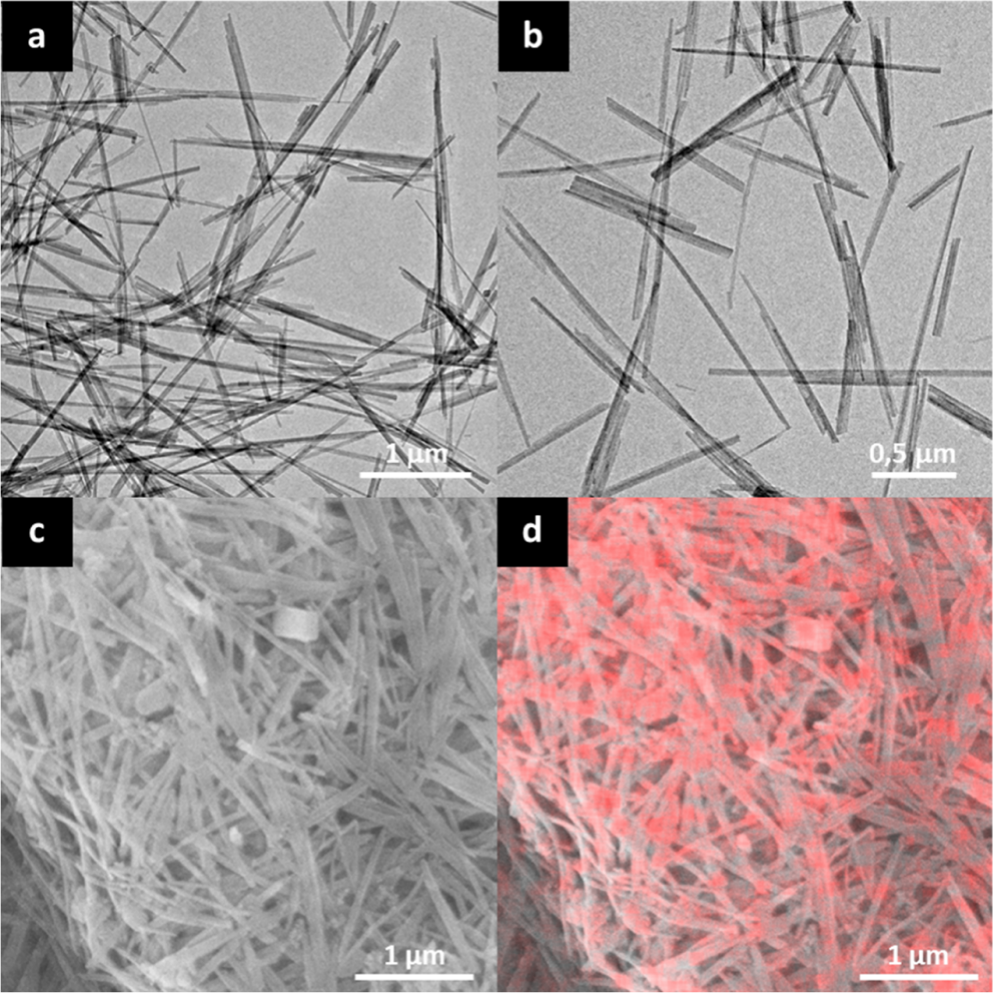
(a,b) TEM micrographs of pristine Sep-OH fibers; (c) SEM micrograph of Sep-OH@CINN-APTES nanofibers and (d) corresponding EDX C elemental map.

To prove the consistency and versatility of the proposed strategy, the surface modification of naturally occurring pretreated sepiolite (Sep-OH) fibers (TEM in Figure 6a,b) (40–150 nm width and 1–10 μm), high BET specific surface area (253 ± 2 m^2^ g^–1^), was also accomplished.

As for SiO_2_@CINN-APTES NPs, the functionalization of Sep fibers with CINN-APTES was first inspected by SEM–EDX (Figure 6c,d). A homogeneous distribution of carbon atoms (red dots in Figure 6d) on Sep-OH@CINN-APTES surfaces was observed, probing the successful grafting of CINN-APTES on the substrate.

**Figure 7:**
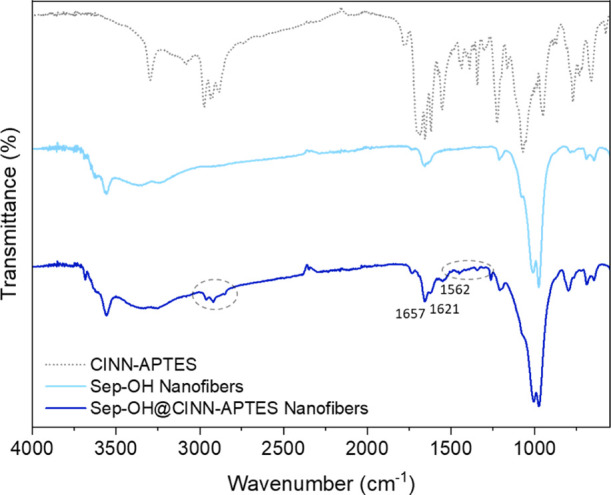
ATR-FTIR spectra of CINN-APTES, Sep-OH nanofibers, and Sep-OH@CINN-APTES nanofibers.

The presence of CINN-APTES at the surface of Sep-OH fibers was further verified by ATR-FTIR spectroscopy (Figure 7). In detail, the following bands appear in the spectrum of Sep-OH@CINN-APTES: the stretching of C–H in the region between 3000 and 2900 cm^–1^ ascribable to the cinnamic group; the stretching vibrations of the carbonyl group (C=O) of the amide functionality at 1657 cm^–1^; and of C=C conjugated to an aromatic ring at 1621 cm^–1^. In addition, the band at 1562 cm^–1^ related to the N–H bending mode of the amide group and the peaks between 1550 and 1350 cm^–1^ belonging to the C–C stretching vibrations of the aromatic ring of CINN-APTES is also clearly detectable.

TGA and CHNS analyses (see eqs S1 and S2 of Supporting Information) allowed us to quantitatively estimate the functionalization degree, which resulted 3.13 and 3.02 no. molecules/nm^2^, respectively.

These outcomes confirm the remarkable value and the flexibility of the methodological protocol adopted in providing tailored silica and silicate NPs with chromophore tails.

Finally, a preliminary proof of concept of the photoreversible behavior of the SiO_2_@CINN-APTES system has been carried out by monitoring the change of UV-DRS spectra of the sample film (see Experimental Section) after irradiation at λ ≥ 365 nm for 24 h (i.e., photodimerization reaction) and, successively, under UV irradiation at λ = 254 nm for 48 h (i.e., photocleavage reaction).

**Figure 8:**
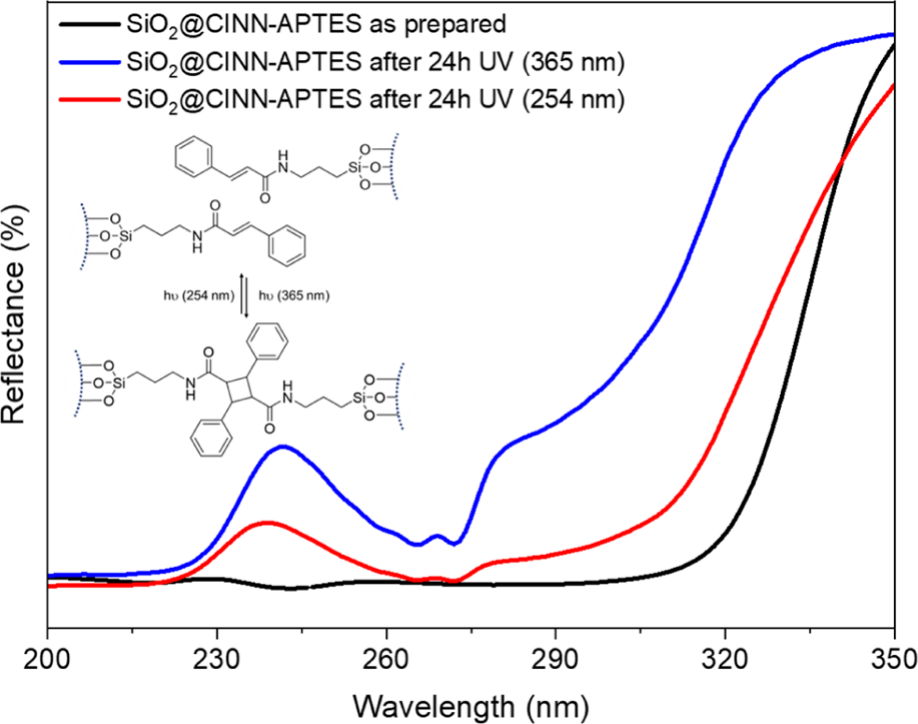
UV-DRS spectra of SiO_2_@CINN-APTES sample: pristine material (black curve); after irradiation at λ ≥ 365 nm for 24 h (i.e. photodimerization, blue curve); SiO_2_@CINN-APTES under successive UV irradiation at λ = 254 nm for 48 h (i.e. photocleavage, red curve). Inset: potential mechanism of photoreversible dimerization of the cinnamic units anchored on SiO_2_ NPs.

The results are summarized in Figure 8.

As expected, the conversion of vinyl aryl links of CINN-APTES units to cyclobutane significantly affects photophysical properties of SiO_2_@CINN-APTES NPs. The diffuse reflectance spectrum of the pristine powders (Figure 8, black curve) reveals an absorption edge at ∼320 nm that upon UV irradiation at 365 nm, it is blue-shifted to ∼270 nm (Figure 8, blue curve) as a result of the breaking of π-conjugation and formation of the cyclobutane links (see inset in Figure 8).^35^ This structural change is also corroborated by the remarkable increase of the reflectance intensity, that is, depletion of the absorption, as testified also by the appearance of more defined bands upon UV irradiation.

Remarkably, after UV exposure at 254 nm, a red shift and a partial depletion of the reflectance intensity, that is, increase of the absorption, is detected, indicating the reversibility of the process through the photocleavage of the dimeric species (Figure 8, red curve). However, the limited recovery to the initial spectral features indicates an incomplete regeneration of the pristine cinnamic structure. This can be explained considering that on the NPs an equilibrium between photodimerization and photocleavage occurs as well as referring to a possible photoinduced cluster formation,^17,36^ which shields from irradiation the cinnamic cross-links positioned between NPs.

## 4 Conclusions

This work proposes an original synthetic strategy for the decoration of silica and sepiolite surfaces with a trialkoxysilane covalently derivatized with cinnamic acid for producing innovative multifunctional hybrid NPs. Upon assessment of the synthesis of the novel organosilane (CINN-APTES), the new molecule was utilized to functionalize the surface of silica and sepiolite NPs and the functionalization degree was estimated by TGA and CHNS analyses. The results point out a much better functionalization efficacy by the one-step route compared to the double-step one (grafting with APTES and subsequent modification with the photoreversible moieties). This suggests that the direct use of CINN-APTES prevents the usual drawbacks associated with the post-grafting of silica surfaces already pre-functionalized with APTES, that is, possible hydrolysis and decomposition before further modification and significant presence of unreacted amino groups, which could impart instability to the system. A preliminary proof of concept of the photoreversibility of the obtained SiO_2_@CINN-APTES NPs has been carried out through UV-DRS, assessing the peculiar light-triggered behavior of the filler system. The overall outcomes indicate the efficacy of the methodological protocol adopted in providing tailored silica and silicate NPs with photoreversible tails, which can be potentially used either in drug release applications or as building blocks for innovative organic–inorganic hybrid nanomaterials with peculiar self-healing or recyclability properties.

## Supporting Information Available

NMR characterization on the reaction products, ATR-FTIR of CINN before and after photodimerization/photocleavage processes and UV−vis photodimerization of CINN units, solution-phase UV−vis normalized spectra of CINN-NHS and CINN-APTES, quantitative analysis of the 29Si MAS spectra, double-step functionalization procedure, and equations for the determination of functionalization degree (PDF)
