# Supplementary material for: Synthesis and Characterization of Alkoxysilane-Bearing Photoreversible Cinnamic Side Groups: A Promising Building-Block for the Design of Multifunctional Silica Nanoparticles
Source: Langmuir. 2022 Dec 8;38(50):15662–71. doi: 10.1021/acs.langmuir.2c02472 (PMC9776512; doi:10.1021/acs.langmuir.2c02472)
Supplement: Supplementary file 1 — la2c02472_si_001.pdf [file la2c02472_si_001.pdf]

Synthesis and characterization of alkoxysilane bearing photoreversible cinnamic side groups: a promising building-block for the design of multifunctional silica nanoparticles

Sara Fernanda Orsini[1], Laura Cipolla[2], Simona Petroni[2], Sandra Dirè[3], Riccardo Ceccato[4], Emanuela Callone[3], Roberta Bongiovanni[5,6], Sara Dalle Vacche[5,6], Barbara Di Credico[1], Silvia Mostoni[1], Roberto Nisticò[1], Luisa Raimondo[1], Roberto Scotti[1,6] and Massimiliano D'Arienzo[1]*

[1] Department of Materials Science, University of Milano-Bicocca, Via R. Cozzi 55, 20125 Milano, Italy

[2] Department of Biotechnology and Biosciences, University of Milano-Bicocca, P.za della Scienza 2, 20126 Milano, Italy

[3] "Klaus Müller" Magnetic Resonance Lab., Department of Industrial Engineering, University of Trento, Via Sommarive 9, 38123 Trento, Italy

[4] Department Industrial Engineering, University of Trento, Via Sommarive 9, 38123 Trento, Italy

[5] Department of Applied Science and Technology, DISAT, Politecnico di Torino, Corso Duca degli Abruzzi 24, 10129 Torino, Italy

[6] Consorzio Interuniversitario Nazionale per la Scienza e Tecnologia dei Materiali, (INSTM) Via G. Giusti, 9, 50121 Firenze, Italy

* massimiliano.darienzo@unimib.it

SUPPORTING INFORMATION

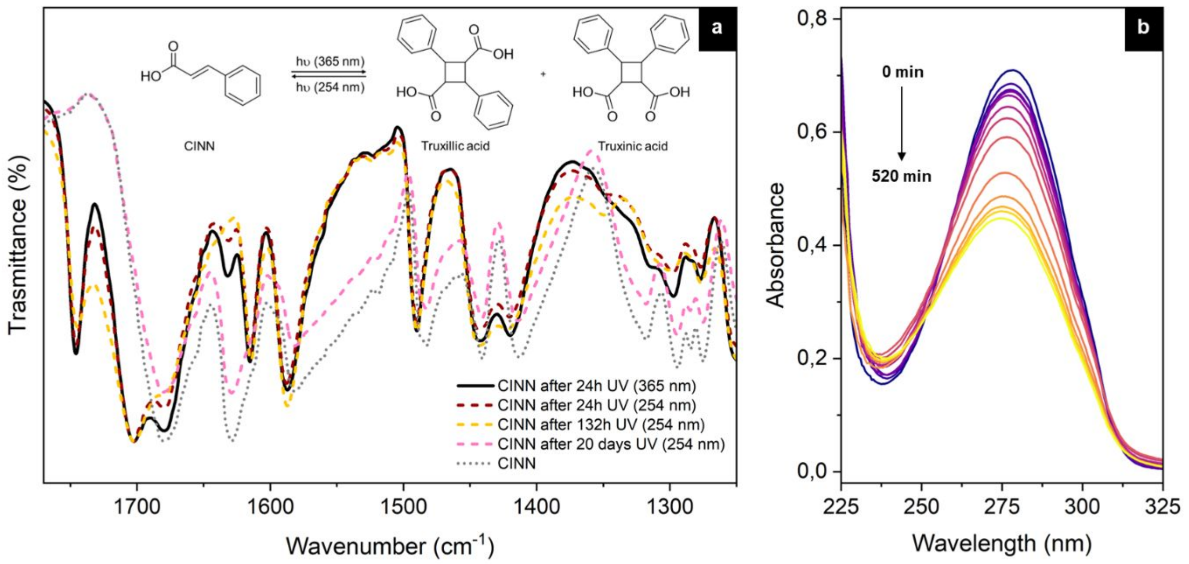

Figure S1. a) ATR-FTIR of CINN before and after photodimerization/photocleavage processes; b) photodimerization of CINN units in chloroform solution.

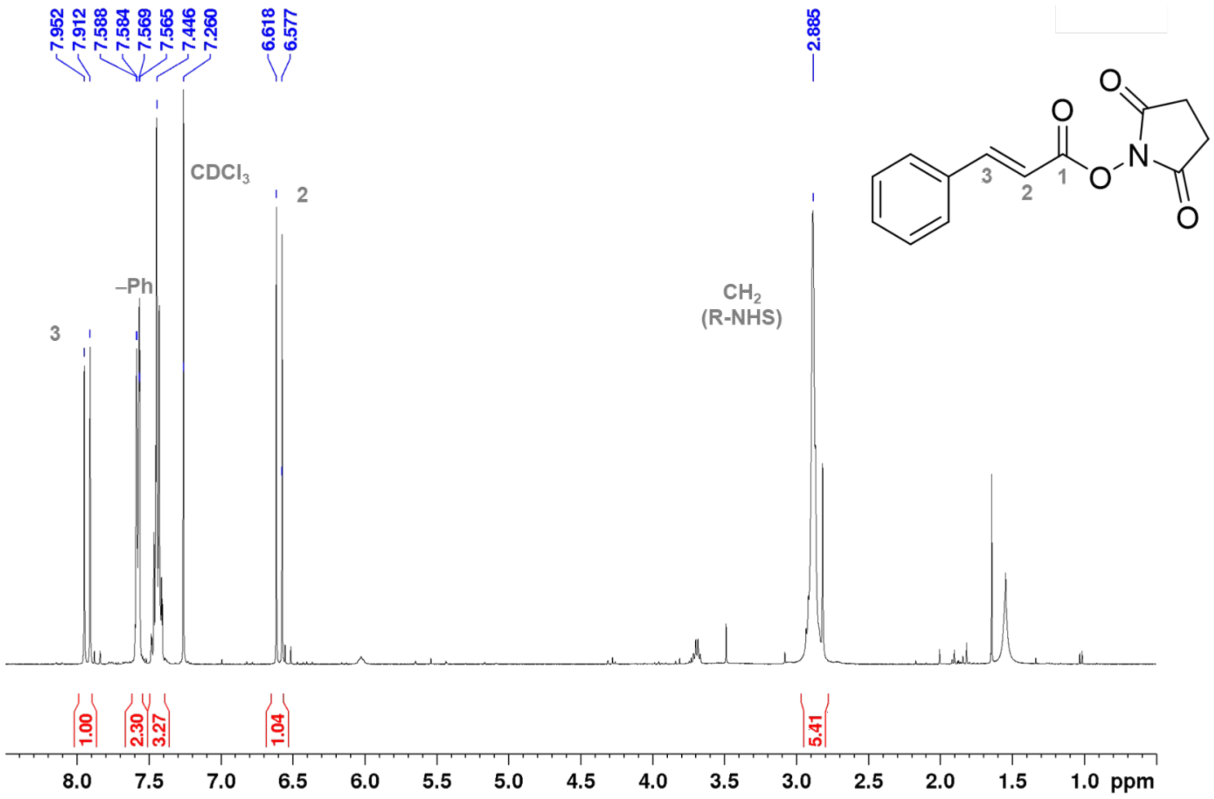

Figure S2. ^{1}H-NMR of CINN-NHS

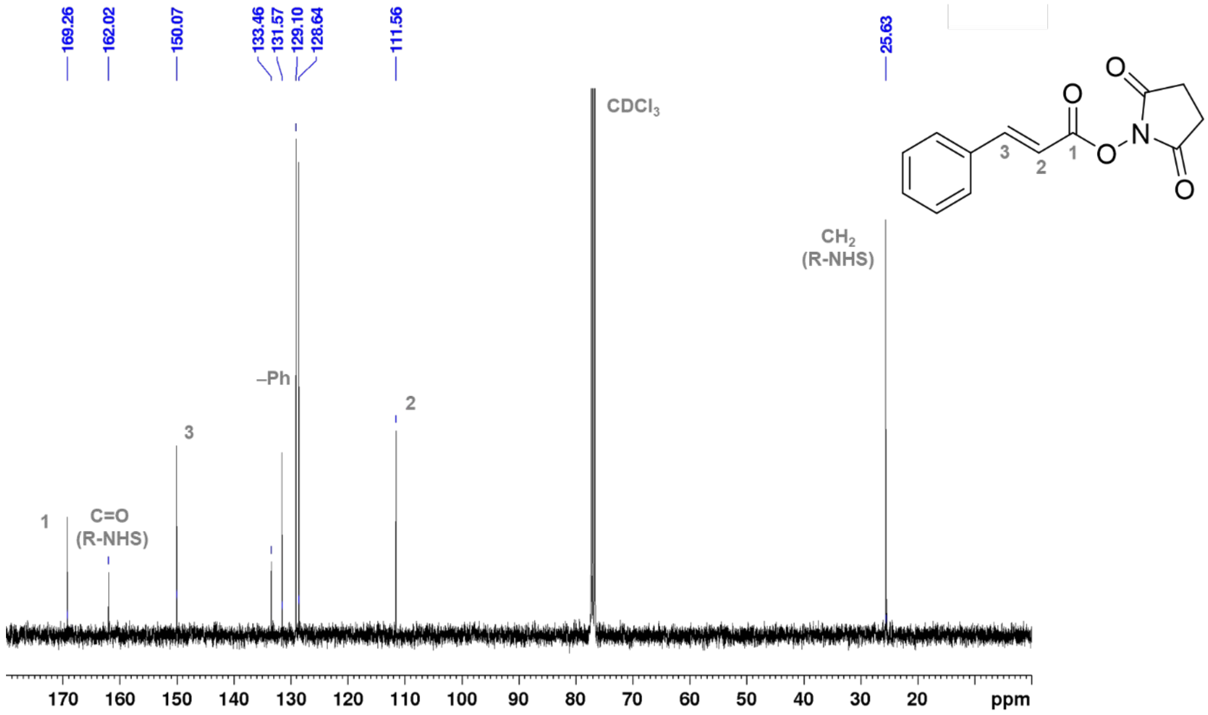

Figure S3. ^{13}C-NMR of CINN-NHS

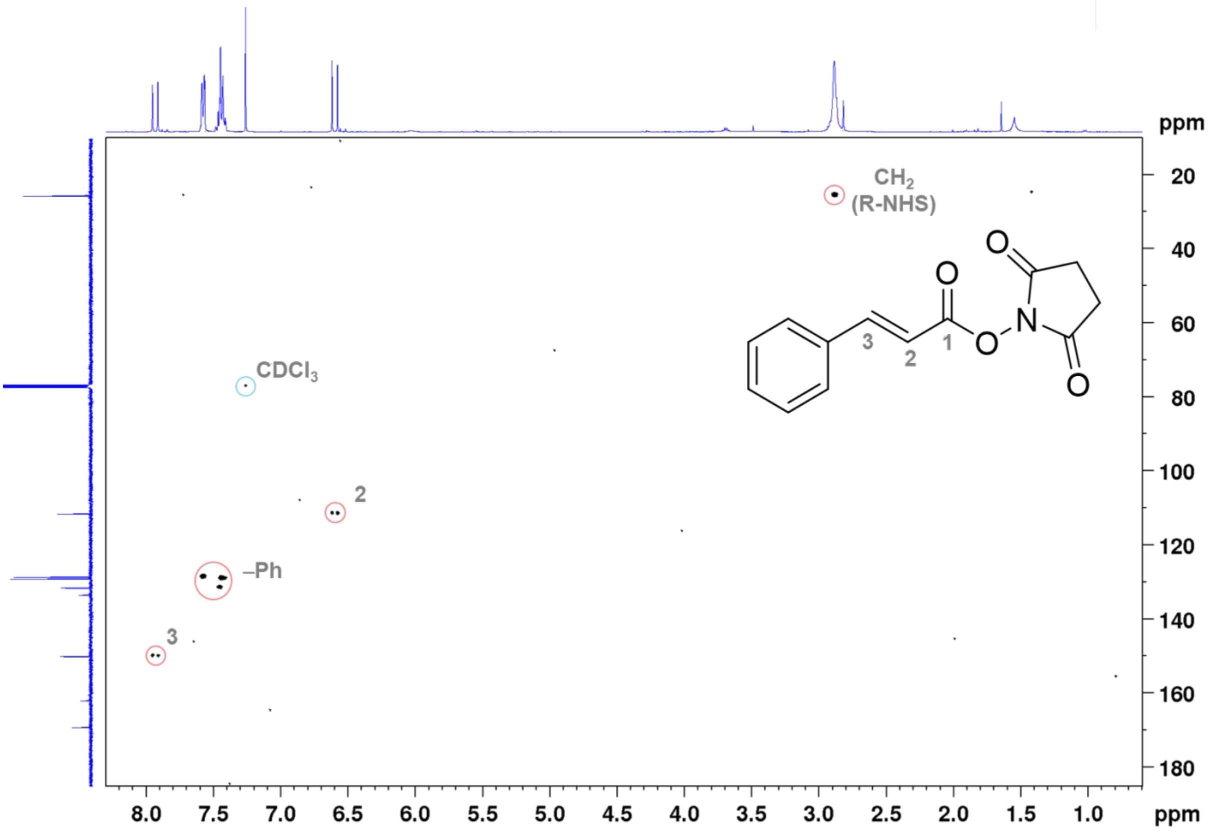

Figure S4. ^{1}H-^{13}C HSQC-NMR of CINN-NHS

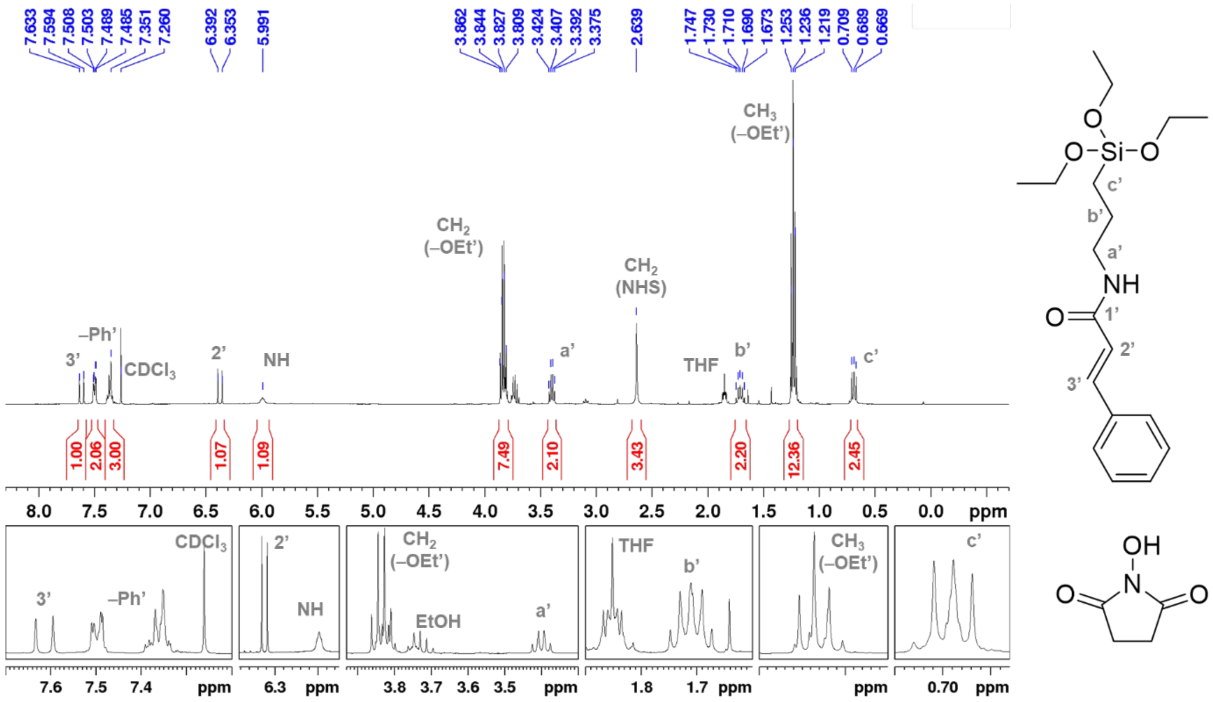

Figure S5. ^{1}H-NMR of CINN-APTES

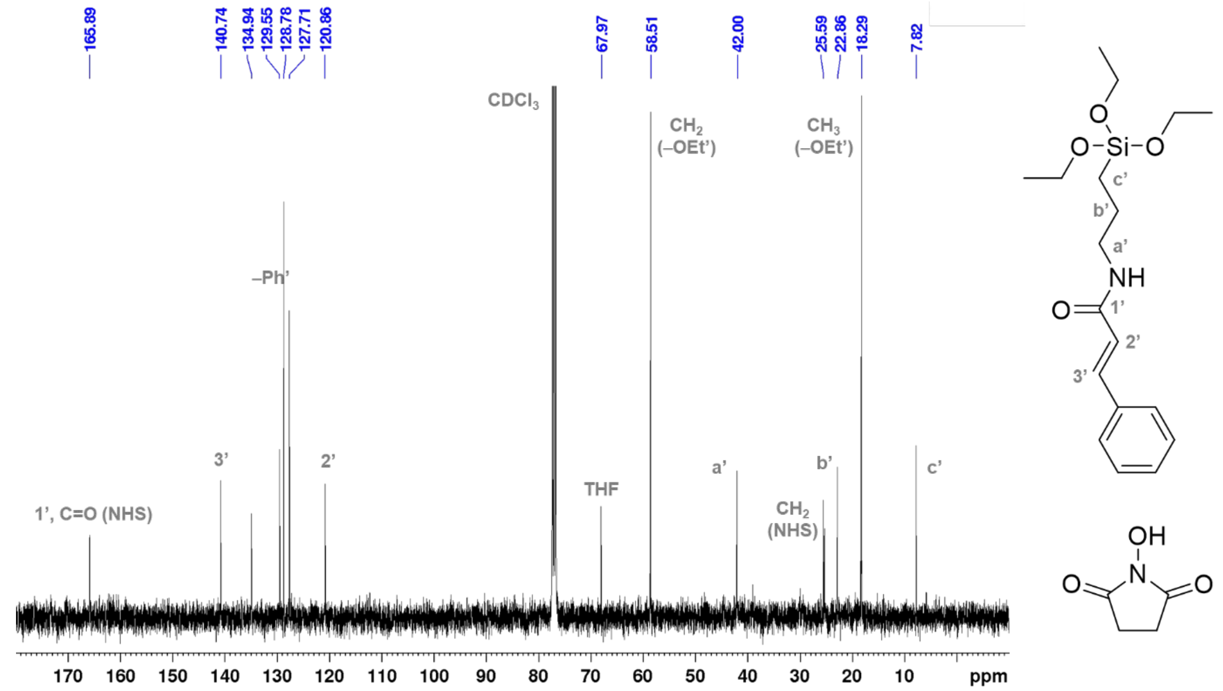

Figure S6. ^{13}C-NMR of CINN-APTES

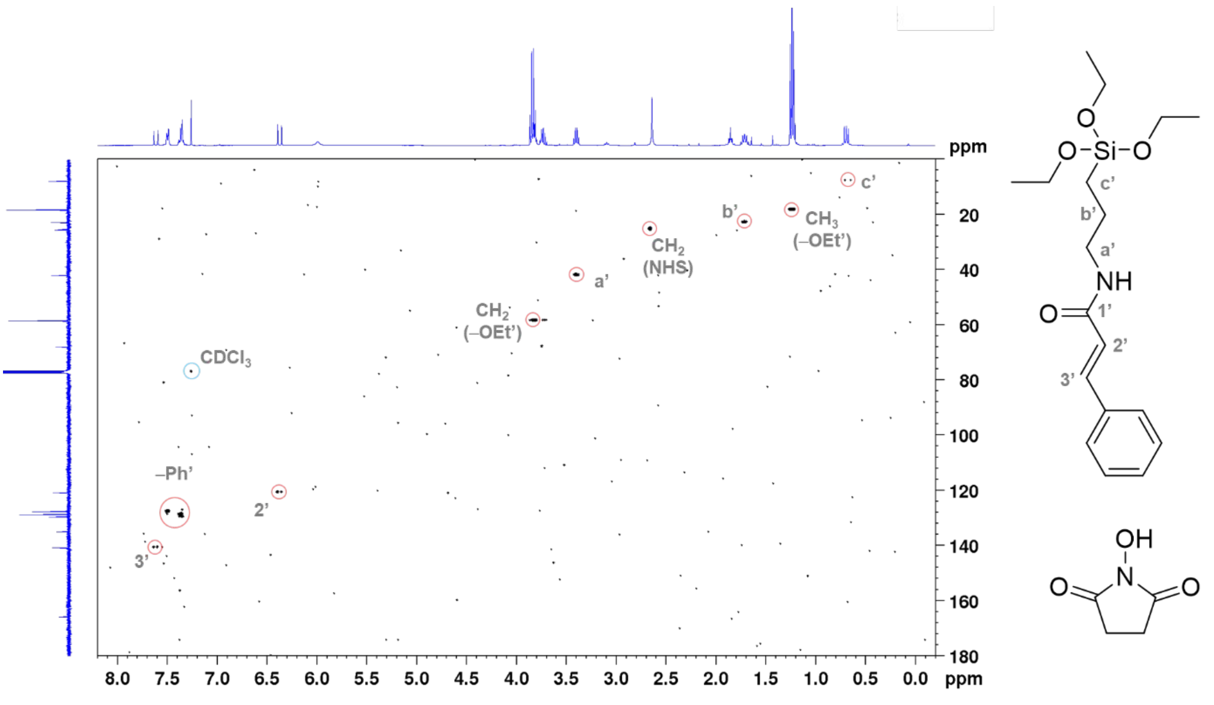

Figure S7. ^{1}H-^{13}C HSQC-NMR of CINN-APTES

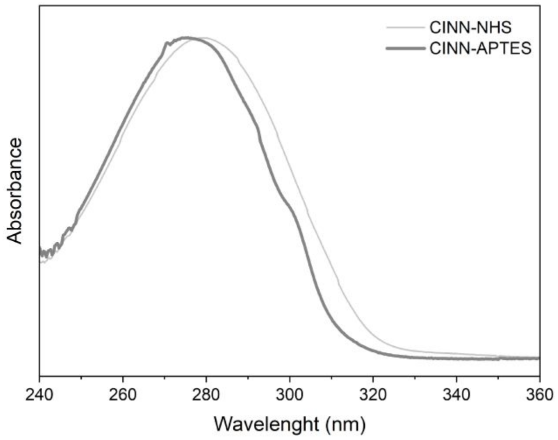

Figure S8. Solution-phase UV-Vis normalized spectra of CINN-NHS (light grey line) and CINN-APTES organosilane (dark grey line). Both spectra have been corrected for solvent absorption.

Table S1. Quantitative analysis of the ^{29}Si MAS spectra of the samples

	T^2	T^3	Q^2	Q^3	Q^4	DOC
**δ (ppm)**	-57.0	-66.3	-90.4	-100.4	-110.0	
**SiO_2 NPs**	-	-	3.8	30.4	65.8	90.5
**SiO_2@CINN-APTES NPs**	1.3	4.6	0.8	17.5	75.8	94.8

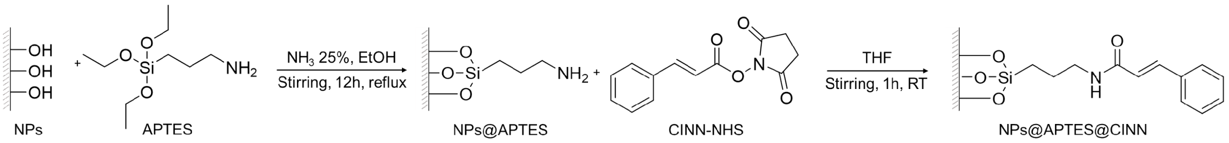

Scheme S1. Double-step functionalization procedure to obtain NPs@CINN@APTES

Functionalization degree of functionalized NPs

The total amount of CINN-APTES grafted onto SiO_2 corresponds to ~ 12.8 wt.% and it was calculated by equation 1:

$$wt.\%(CINN-APTES) = \frac{\Delta wt.\%_{(150-1000°C)st} - \Delta wt.\%_{(OH+OEt)st}(NPs)}{MW(CINN-APTES) - \frac{MW(H_2O)}{2}} MW(CINN-APTES) \quad (1)$$

considering the net weight loss of SiO_2@CINN-APTES between 150 and 1000 °C ($\Delta wt.\%_{(150\text{-}1000°C)st}$), the net weight loss of naked silica NPs between 150 and 1000 °C ($\Delta wt.\%_{(OH+Oet)st}$(NPs)), the molecular weight of the CINN-APTES without the silicon head (MW(CINN-APTES)), and the molecular weight of water. This equation considers the hydroxyl groups involved in the functionalization bond and the hydrolysed and not reacted hydroxyl groups of CINN-APTES. CINN-APTES is assumed to be grafted onto silica NPs with two of the three ethoxy groups. From the value of wt.%(CINN-APTES), the number of molecules of organosilane grafted per square nanometre (σ) was calculated, which corresponds to 1.82 n. molecules/nm^2 (equation 2)

$$\sigma = \frac{\frac{wt.\%\,(CINN-APTES)}{MW(CINN-APTES)} \cdot N_A}{wt.\%_{(1000°C)st} \cdot A_{sup}(NPs)} \quad (2)$$

Eq. 1 and 2 were used to also estimate the functionalization degree of SiO_2@APTES, SiO_2@APTES@CINN NPs and Sep-OH@CINN-APTES Nanofibers.
